# Impact of COVID-19 on chronic kidney disease progression in non-dialysis patients: a retrospective cohort study in Palestine

**DOI:** 10.1080/07853890.2025.2479236

**Published:** 2025-03-18

**Authors:** Fadi Basha, Yazan Dumaidi, Masa Sabbobeh, Hamzeh Almasri, Ahmad Rjoub, Zakaria Hamdan, Zaher Nazzal

**Affiliations:** a Department of Medicine, Faculty of Medicine and Health Sciences, An-Najah National University, Nablus, Palestine; b Consultant Nephrology, Al Watani Hospital Department of Nephrology, Ministry of Health, Nablus, Palestine; c Consultant Internal Medicine, Internal Medicine Department, An-Najah National University Hospital, Nablus, Palestine; d Consultant Community Medicine, Department of Medicine, Faculty of Medicine and Health Sciences, An-Najah National University, Nablus, Palestine

**Keywords:** Chronic kidney disease, COVID-19, eGFR, non-dialytic patients

## Abstract

**Background:**

This study aims to evaluate the impact of COVID-19 on the progression of CKD in non-dialysis patients and its relation to clinical outcomes in Palestine.

**Materials and methods:**

We conducted a retrospective cohort study that followed non-dialysis CKD patients receiving treatment at outpatient clinics in governmental hospitals. Out of the 248 CKD patients who met the inclusion criteria, 98 were diagnosed with COVID-19 between March 2020 and March 2022. We collected data at three distinct time intervals, both prior to and after their COVID-19 infection. We examined the decline in eGFR and gathered demographic information, hospitalization, and mortality rates. The drop in eGFR was recorded 15 months from baseline.

**Results:**

The mean age of the patients was 55 years, with 55.6% being male. Patients diagnosed with COVID-19 faced a significantly higher risk of rapid deterioration in eGFR, with a 3.7-fold increase compared to those without COVID-19 (a*p*-value: <0.001; aOR: 3.7; 95% CI: 2.1–6.3). Additionally, COVID-19 patients had 4.4 times higher mortality rates (a*p*-value: 0.005; aOR: 4.4; 95% CI: 1.6–12.4), 13.3 times higher rates of dialysis initiation within 15 months post-baseline (a*p*-value: <0.001; aOR: 13.3; 95% CI: 6.1–28.7), and 3.5 times higher rates of hospital admissions (a*p*-value: <0.001; aOR: 3.5; 95% CI: 1.8–6.7) compared to the COVID-19 negative group.

**Conclusion:**

CKD patients who contract COVID-19 experience a more rapid decline in kidney function, leading to worse health outcomes, including increased mortality rates, a greater need for dialysis, and higher hospitalization rates.

## KEY MESSAGES

- Patients with CKD who contract COVID-19 experience a rapid decline in kidney function, which increases their need for on dialysis.
- COVID-19-infected individuals with CKD exhibit an elevated likelihood of being hospitalized, requiring ICU admission, respiratory support, and a heightened mortality rate.

## Introduction

Chronic Kidney Disease (CKD) is a common disease worldwide with a prevalence of 13.4% [1,2]. The incidence of CKD has been on the rise, impacting around 843.6 million people globally in 2017 [3]. Findings from the Global Burden of Disease studies indicate that CKD has become a significant contributor to global mortality [2,4]. Diagnosis is established via the levels of estimated glomerular filtration rate (eGFR), an important indicator of kidney function [5]. CKD is defined as a reduction in renal function manifested by an eGFR of less than 60 mL/min per 1.73 m^2^, indications of kidney damage, or both, lasting at least three months [6].

CKD can be caused by many systemic diseases, such as autoimmune diseases, diabetes, and chronic infection [7]. A decline in the eGFR as a sign of progression of CKD has been shown to be associated with acute infections [8], such as H1N1 and COVID-19 [9,10]. COVID-19 has had an extraordinary impact on humanity, with widespread global transmission and a startling number of people affected. The WHO estimated that 769 million individuals had contracted the virus by August 2023, with over 6.9 million deaths [11,12].

Multiple studies have demonstrated an association between CKD and COVID-19 since the pandemic’s onset, indicating that patients who contracted COVID-19 while having CKD had higher morbidity and mortality than non-CKD populations [10,13,14]. Clinical complications ranged from pneumonia to increased need for oxygen support and higher levels of in-hospital neutrophilia, in addition to dialysis or renal transplant. COVID-19 has also been associated with deteriorating renal function parameters in patients who received COVID-19 after their hospitalization [14–17].

Upon review of the available literature, we have observed that a potential correlation between COVID-19 and CKD has been studied in different studies and settings. Nevertheless, a thorough analysis of the correlation between COVID-19 and the progression of CKD in non-dialysis patients, which looks into the rate of change in GFR, in addition to the presence of rapid eGFR deterioration, has yet to be conducted. Studying this population will improve understanding of the effects of COVID-19 on moderate to severe CKD and help prevent deterioration into renal replacement therapy, which in turn will help reduce the burden of this disease.

This study aimed to evaluate the effect of COVID-19 infection on the progression of CKD in non-dialysis patients. We hope that the knowledge produced by this research will aid in establishing guidelines for managing CKD patients who are infected with COVID-19 and any related future viral respiratory outbreaks by providing valuable insights for treating non-dialytic CKD patients in different healthcare environments and settings.

## Materials and methods

### Study design and participants

We conducted a retrospective cohort study that followed CKD non-dialysis patients to evaluate the effect of COVID-19 on kidney function and clinical outcomes. The study population was CKD patients not requiring dialysis at the start of the period who had encountered COVID-19 infection, with the control group being CKD patients who did not have COVID-19. The study was carried out at the outpatient clinics of Al-Watani Hospital in Nablus, Thabet Thabet Hospital in Tulkarem, and Jenin Hospital in Jenin. These hospitals jointly function as the central government healthcare settings responsible for providing healthcare services to patients suffering from CKD in the northern West Bank region. The study included only these cities, primarily because of logistical challenges associated with travel between other areas, due to the political situation in Palestine. Moreover, given the relatively small and homogenous Palestinian population, characterized by limited diversity, key variables including ethnicity, race, access to healthcare, socioeconomic status, and employment levels were notably similar across the studied cities. This similarity enhances the potential for generalizing the study results to broader populations.

The study was conducted between March 2020 and March 2022, with all COVID-19 testing being within this period. We took measures to include both COVID-19-positive and COVID-19-negative patients by ensuring that each patient had at least one record (either positive or negative) in the national COVID-19 testing system. The follow-up period was 15 months on average between the first and last data entries. The inclusion criteria covered patients meeting the following conditions: (1) having eGFR below 60 ml/s at the beginning of the specified timeframe, (2) not requiring regular dialysis, and (3) being between 18 and 75 years old. Additionally, for the COVID-19-positive cohort, positive test results needed to be documented between March 2020 and March 2022. On the other hand, exclusion criteria included patients requiring regular dialysis and those with incomplete medical records.

Ethical approval was obtained from the institutional review board (IRB) at An-Najah National University (Reference #: Med. August 2022/35) and the Palestinian Ministry of Health (MoH), with total commitment to patient confidentiality. The IRB granted a waiver for informed consent since the research relied on secondary data. This data was securely stored on password-protected computers, ensuring that only the research team had access to it, and its utilization was exclusively for research objectives. We removed any identifying information such as names, addresses, and other personally identifiable information.

### Data collection

The data collection process included three central hospitals located in three primary cities in the northern West Bank of Palestine. In Palestine, governmental hospitals adhere to an unified data registry system for patient information, facilitated through a standardized computerized system overseen by the Palestinian MoH. This centralized approach ensures consistency across all governmental hospitals in the region. Additionally, the history of COVID-19 infection was gathered through the MoH’s dedicated online portal, which serves as a common platform utilized by all hospitals. For each subject, we recorded three sets of data: the baseline data entry consisting of background and clinical variables taken from patient records before testing positive for COVID-19. The patients were followed-up for a total of 15 months. Each set of data included the patient’s COVID-19 testing history, outcome variables such as mortality during the follow-up period, hospitalization due to medical causes, ICU admission, respiratory support defined as the need for ventilator, dialysis, and kidney function tests (eGFR & Creatinine). Additionally, background variables such as age, gender, the date of each data entry, and past medical history (cardiovascular diseases (CVD), hypertension (HTN), and diabetes mellitus (DM)) were collected. The baseline and follow-up data were collected from patient’s medical records in the three targeted governmental hospital clinics.

History of COVID-19 infection was collected via the MoH’s COVID-19 online portal, using patient ID numbers to collect previous positive tests. The portal is dependent on PCR COVID-19 tests performed by the MoH laboratories. Each patient had 3 COVID-19 tests on average, regardless of the result. The eGFR was calculated via the National Kidney Foundation eGFR calculator, using the CKD-EPI creatinine equation, with serum creatinine, age, and gender being taken into equation and standard assays without adjusting for the body surface [13]. Related to eGFR was also the rapid eGFR deterioration, defined as the sustained decline in eGFR of >5 ml/min/year [14]. The use of CKD-EPI originated from its suitability for the available creatine data, as well as the lack of racial mention in the health system utilized in the collection, making it difficult to employ different formulas. Additionally, CKD-EPI has a 98% specificity for identifying eGFR <60 ml/min per 1.73 m^2^ [18].

### Statistical analysis

The Statistical Package of Social Science (SPSS) version 26.0 and Microsoft Excel software were used for data entry and analysis in this study. Demographic characteristics and laboratory variables of the sample were displayed through tables and figures, providing percentages, means, and standard deviations as needed. We performed bivariate analysis utilizing the chi-square test and independent *t*-test to assess the differences between groups. Furthermore, we conducted multivariate analysis using binary logistic regression model for each outcome to the evaluate the influence of COVID-19 on various study outcomes, while adjusting for potential confounding variables. The variables incorporated into the model included eGFR at baseline, age, gender, DM, HTN, and CVD. Multivariate analysis results were presented as adjusted *p*-values (a*p*-values), adjusted odds ratios (aORs), and their corresponding 95% confidence intervals (CIs). The significance level defined in this study is *p* value ≤0.05.

## Results

**Figure 1.**
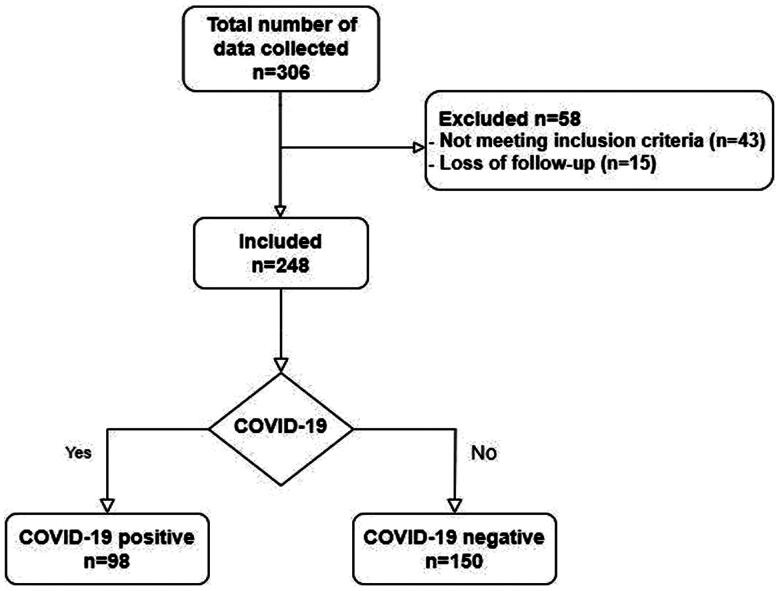
Flowchart of the study.

A total of 306 patients were included in the study, of whom 58 were excluded. Consequently, 248 patients satisfied the established criteria for inclusion and were subsequently enrolled in the study. The 58 patients excluded from the initial sample were removed for either loss of follow-up in nephrology clinics and incomplete data entry (15 patients) or failure to meet the inclusion criteria (43 patients). This lack of follow-up can be attributed to factors such as passing or hesitance to attend health clinics for regular check-ups during the pandemic. Of the included patients, 150 were COVID-19 negative, and 98 were COVID-19 positive (Figure 1).

### Baseline characteristics

The mean age of the patients was 55 years, with no significant difference between the positive and negative groups. Regarding the patient’s gender, 55.6% were male and 44.4% were female. Several patients in the study had medical comorbidities, with 103 patients having diabetes mellitus, 126 having hypertension and 53 having cardiovascular disease, with no significant difference between the two groups (Table 1).

**Table 1.** Baseline characteristics of the chronic kidney disease patients (*n* = 248).

		COVID-19		
Item	Total frequency (%)	Positive frequency (%)	Negative frequency (%)	*p*-Value*
Overall	248 (100%)	98 (39.5%)	150 (60.5%)	**--**
Age (Mean ± SD)	55 (±15.6)	56.6 (±13.9)	53.9 (±16.5)	0.179
Gender				
Male	138 (55.6%)	57 (58.2%)	81 (54%)	0.519
Female	110 (44.4%)	41 (41.8%)	69 (46%)	
Diabetes mellitus	103 (41.5%)	41 (41.8%)	62 (41.3%)	0.973
Hypertension	126 (50.8%)	48 (49.0%)	78 (52.0%)	0.642
Cardiovascular disease	53 (21.4%)	17 (17.3%)	36 (24.0%)	0.211
Baseline eGFR (Mean ± SD)	39.9 *±* 15.2	37.9 ± 16	41.4 ± 14.6	0.077
Baseline creatinine (Mean ± SD)	2.2 *±* 1.64	2.47 ± 2.06	2.04 ± 1.29	0.067

* Pearson Chi-square test and independent *t*-test.

### Kidney functions and COVID-19

**Figure 2.**
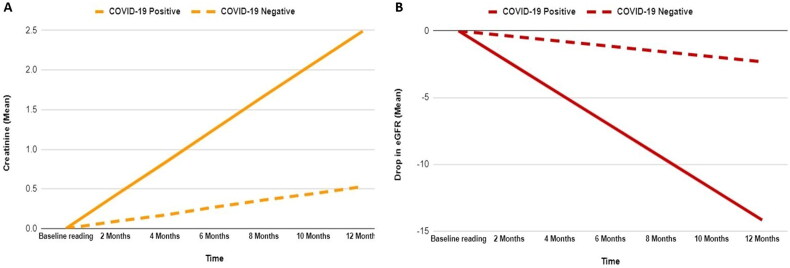
Monthly changes in kidney function indicators, creatinine (A), and eGFR (B) in patients with chronic kidney disease with COVID-19.

Table 2 and Figure 2 compare kidney function results between COVID-19-positive and negative patients after a 15-month follow-up. There was a significant increase in serum creatinine levels between the COVID-19 positive and negative groups from baseline to final measures (1.33 vs. 0.64, *p* = 0.001). This increase was reflected in the higher monthly rise in serum creatinine for the COVID-19 positive group compared to the COVID-19 negative group (0.21 vs. 0.04, *p* = 0.001) (Figure 2(A)). For the eGFR values, COVID-19-positive patients had a larger decrease between baseline and last readings (9.46 ± 13.88) than COVID-19-negative patients (3.15 ± 11.7), with a p-value of 0.001. This was also reflected in a higher monthly reduction in eGFR in the COVID-19 positive group compared to the COVID-19 negative group (1.18 vs. 0.19, *p* < 0.001) (Figure 2(B)).

**Table 2.** Comparison of kidney function tests between COVID-19 positive and COVID-19 negative CKD patients.

Variable	COVID-19 positive (Mean ± SD)	COVID-19 negative (Mean ± SD)	*p*-Value*
eGFR difference between baseline and last readings	9.46 ± 13.88	3.15 ± 11.7	<0.001
Creatinine difference between baseline and last readings	1.33 ± 2.3	0.64 ± 1.7	0.006
Deterioration in eGFR per month	1.18 ± 2.78	0.19 ± 0.89	<0.001
Increase in creatinine per month	0.21 ± 0.52	0.04 ± 0.13	<0.001

* Independent *t*-test.

### Rapid eGFR deterioration

The relationship between COVID-19 and rapid eGFR decline was investigated. COVID-19-positive patients had a significantly higher percentage of rapid eGFR deterioration than COVID-19-negative patients (62.2 vs. 32%, *p*-value: 0.001). Multivariate analysis revealed that CKD patients infected with COVID-19 have a 3.7 times higher risk of rapid eGFR deterioration compared to the non-COVID-19 group (a*p*-value: <0.001, aOR: 3.7, 95% CI: 2.1–6.3). Other variables, such as age, diabetes, gender, cardiovascular disease, and hypertension, did not reveal a significant association with rapid eGFR deterioration (Table 3).

**Table 3.** Rapid eGFR deterioration rates among different patient groups.

	Rapid eGFR deterioration			Multivariate analysis	
	Yes (%)	No (%)	*p*-Value*	aOR (95% CI)	a*p*-Value
COVID-19					
Yes	61 (62.2%)	37 (37.8%)	<0.001	3.7 (2.1–6.3)	<0.001
No^†^	48 (32.0%)	102 (68.0%)			
Age					
≤55 years	46 (45.0%)	56 (55.0%)	0.761	1.3 (0.6–2.1)	0.646
>55 years^†^	63 (43.2%)	83 (56.8%)			
Gender					
Male^†^	58 (42.0%)	80 (58.0%)	0.494	1.2 (0.7–2.1)	0.422
Female	51 (46.4%)	59 (53.6%)			
DM					
Yes	44 (42.7%)	59 (57.3%)	0.742	1 (0.6-1.9)	0.910
No^†^	65 (44.8%)	80 (55.2%)			
CVD					
Yes	21 (39.6%)	32 (60.4%)	0.474	1.2 (0.6-2.5)	0.606
No^†^	88 (45.1%)	107 (54.9%)			
HTN					
Yes	60 (47.6%)	66 (52.4%)	0.273	0.6 (0.4–1.1)	0.122
No^†^	49 (40.1%)	73 (59.9%)			
Baseline eGFR	39.7 ± 14.5	40.2 ± 15.7	0.804	3.7 (2.1–6.3)	0.765

* Pearson Chi-square test; aOR**:** adjusted OR; aP-value: adjusted *p*-value.

^†^ Reference group; **the results are adjusted for age, gender, DM, HTN, CVD, and eGFR at baseline.

### Clinical outcomes

Table 4 presents the bivariate and multivariate analysis results on the relation between COVID-19 status and different clinical outcomes, including mortality, hospital admission, ICU admission, respiratory support, and the need for dialysis 15 months after the baseline reading. Binary logistic regression model was employed for each outcome, adjusting for potential confounding variables such as eGFR at baseline, age, gender, DM, HTN, and CVD. Regarding mortality, the results showed that it was predicted by eGFR at baseline (a*p*-value: <0.001, aOR: 1.07, 95% CI: 1.03–1.10) and COVID-19 status (a*p*-value: 0.005, aOR: 4.4, 95% CI: 1.6–12.4). Similarly, the COVID-19 positive group exhibited a significantly higher risk for patients necessitating hospital admission than the COVID-19 negative group (a*p*-value: <0.001, aOR: 3.5, 95% CI: 1.8–6.7), along with males (a*p*-value: 0.038, aOR: 2.1, 95% CI: 1.1–4.0) and those with higher eGFR at baseline (a*p*-value: <0.001, aOR: 1.1, 95% CI: 1.06–1.20).

**Table 4.** Clinical outcomes of COVID-19 among different patient groups.

	COVID-19		*p*-Value*	Multivariate analysis**	
	Yes (%)	No^†^ (%)		aOR (95% CI)	a*p*-Value
Mortality					
Yes	17 (17.3%)	6 (2.4%)	<0.001	4.4 (1.6-12.4)	0.005
No	81 (82.7%)	144 (97.6%)			
Hospital admission					
Yes	50 (51%)	37 (24.7%)	<0.001	3.5 (1.8–6.8)	<0.001
No	48 (49%)	113 (75.3%)			
ICU admission					
Yes	30 (30.6%	27 (18%)	0.021	1.9 (0.9–3.7)	0.114
No	68 (69.4%)	123 (82%)			
Respiratory support					
Yes	19 (19.4%)	20 (13.3%)	0.200	1.1 (0.5–2.5)	0.764
No	79 (80.6%)	130 (86.7%)			
Need for dialysis 15 months after baseline reading.					
Yes	55 (56.1%)	20 (13.3%)	<0.001	13.3 (6.1–28.7)	<0.001
No	43 (43.9%)	130 (86.7%)			

* Pearson Chi-square test; aOR**:** adjusted OR; a*p*-value: adjusted *p*-Value.

^†^ Reference group; **the results of each outcome are adjusted for age, gender, DM, HTN, CVD, and eGFR at baseline.

Additionally, the COVID-19-positive group showed a significantly elevated risk of requiring dialysis 15 months post-baseline assessment (a*p*-value: <0.001, aOR: 13.3, 95% CI: 6.1–28.7), as did males (a*p*-value: 0.004, aOR: 3.0, 95% CI: 1.4–6.3) and patients with higher eGFR at baseline (a*p*-value: <0.001, aOR: 1.07, 95% CI: 1.04–1.10). However, COVID-19 infection did not predict the risk of ICU admission or the need for respiratory support. Instead, higher eGFR at baseline emerged as the predictor for both outcomes; (a*p*-value: <0.001, aOR: 1.1, 95% CI: 1.07–1.20) and (a*p*-value: <0.001, aOR: 1.09, 95% CI: 1.06–1.12), respectively.

## Discussion

Multiple studies have linked COVID-19 to worsening kidney function, demonstrating increased serum creatinine and urea levels and a subsequent decrease in eGFR [19–21]. Studies are showing that the infection’s impact on immune dysregulation and the impairment of renal perfusion is attributed to the correlations between COVID-19 and kidney impairment [22]. However, the evidence is scant due to the novelty of the disease and the time needed for long-term effects on kidney function to manifest in patients. The study aimed to compare COVID-19 and non-COVID-19 CKD patients regarding their kidney function markers (Urea, Creatinine, eGFR) chronologically and how COVID-19 infection has affected the course of the disease, particularly regarding mortality, rapid eGFR decline, and use of dialysis.

In this study, there was a steeper deterioration in eGFR levels in COVID-19-positive patients compared to COVID-19-negative ones, which is consistent with other studies that confirm the same finding of eGFR decline ≥30% [23–25]. This deterioration was shown to be independent of confounding factors such as age or other comorbidities compared throughout the study, which strongly suggests the effect COVID-19 plays in the prognosis of CKD. In addition to the decline in the kidney function markers, the rate of decline was fast in COVID-19-positive patients, with more than 60% of them having a rapid eGFR decline, stressing the fact that COVID-19 may be linked to a fast deterioration in the symptoms of the patients, which increases morbidity and mortality. Additional kidney markers were taken into account, revealing a monthly rise in Creatinine of 0.21 ± 0.52 among the COVID-19-positive group, surpassing the 0.04 ± 0.13 reported in the COVID-19-negative group.

The study also showed an increased need for dialysis in the COVID-19-positive group compared to the COVID-19-negative group. The need for dialysis was 56.1% for the COVID-19-positive group at 15 months after the baseline reading, while it was 13.3% for the COVID-19-negative group (a*p*-value <0.001). Notably, COVID-19-positive CKD patients were 13.3 times at elevated risk of requiring dialysis 15 months post-baseline compared to the COVID-19-negative group. The need for dialysis was also found to be an independent factor affecting mortality, hospital and ICU admission, and respiratory support, which further explains the link between COVID-19 and mortality rates, as the infection has led to a worsened kidney function, requiring dialysis, and thus leading to a worse outcome for the patients. These findings are consistent with a recent study by Ahmed et al. conducted in Palestine on 1554 dialysis patients with COVID-19, which found that they had higher rates of hospital admission, ICU admission, respiratory support, and mortality compared to the non-dialytic population and the general population [26].

Mortality rates in the COVID-19 positive population were 4.4 times higher than in the COVID-19-negative population, which is also consistent with other studies [16,27,28]. Around 17% of patients in the COVID-19-positive group passed away during the study compared to only 2.4% in the negative group, and this increase was adjusted to age, gender, and other comorbidities that patients had. The mortality rate is favorable compared to a study conducted by Gibertoni et al. on 4716 patients in Italy, where the mortality rate was around 44.6% in the COVID-19 positive population compared to 4.7% in the COVID-19 negative group [16]. A study conducted in Spain by Portolés et al. on 603 Patients showed a mortality rate for the COVID-19-positive group to be around 44% [28]. The sample size difference could explain this. The increased mortality rate in the COVID-19 positive group might be due to the worse course of COVID-19 in these patients due to the CKD, as evidenced also by higher admission rates in the COVID-19 positive group, or it might be due to the long-term sequelae the disease has on kidney function, evidenced by the higher rate of dialysis and dialysis being an independent factor for increased mortality.

Hospital admission rates were much higher in the COVID-19 population, 51%, than in the COVID-19-negative Population, 24.7%, and the increase was independent of age and other comorbidities except for diabetes.

More research is needed to investigate the link between COVID-19 and renal impairment, especially given the geographical demographics targeted in this study. Also, follow-up studies focusing on the same population and sample are needed to address potential long-term effects of COVID-19 on kidney function and other studied variables. Remarkably, this study revealed a substantial clinical link between COVID-19 infection and renal function deterioration, creating early hurdles for healthcare personnel due to a lack of earlier evidence-based guidance on associated viral strains and emerging disorders. This study has been designed to serve as an essential starting point for the literature and guiding protocols that aim to direct healthcare practitioners in their evidence-based practice, underlining the need for early monitoring of kidney markers and their incorporation into the overall clinical management of patients with similar demographic features.

Our study is subject to several limitations, primarily concerning the data collection process. These limitations include incomplete documentation of patients’ medical histories, loss of patient follow-up in nephrology clinics, and uncertainty regarding whether all patients were referred for COVID-19 testing through the Palestinian MoH COVID-19 portal system. To maintain the validity of our findings, we excluded patients with incomplete records.

Although our study may have some limitations, it also has notable strengths that should be acknowledged. Firstly, we utilized a centralized national COVID-19 database, providing us with a reliable and credible source of information that was easily accessible. Additionally, the data collection process was conducted across three different central hospitals in the northern West Bank, covering three main cities in Palestine. This approach enabled us to concurrently analyze multiple variables, thereby distinguishing our research from other similar studies and contributing significantly to the existing literature.

## Conclusions

CKD combined with COVID-19 has been found to be associated with a more rapid decline in kidney function tests, increased rate of mortality, increased need for dialysis, a higher rate of hospital admission, ICU admission and respiratory support. Implementing policies to reduce infection rates is thus warranted to reduce short- and long-term morbidity and mortality in CKD patients, as well as to assist healthcare workers developing clear guidelines regarding the effect of respiratory viruses such as COVID-19 on the prognosis of CKD patients. Furthermore, we suggest conducting additional studies that incorporate a non-CKD control group to provide deeper insights into the variables under examination. Additionally, adopting a multi-centre approach that includes the entire Palestinian healthcare system would be beneficial. Moreover, further studies are warranted to study the link between COVID-19 and CKD in a molecular and immunological approach to understand the relationship between CKD and COVID-19.

## Data Availability

The datasets generated during and analyzed during the current study are available from the corresponding author on reasonable request.
